# Factors associated with acquired Anti IFN- γ autoantibody in patients with nontuberculous mycobacterial infection

**DOI:** 10.1371/journal.pone.0176342

**Published:** 2017-04-24

**Authors:** Pakpoom Phoompoung, Nasikarn Ankasekwinai, Manop Pithukpakorn, Suporn Foongladda, Pinklow Umrod, Bhoom Suktitipat, Surakameth Mahasirimongkol, Sasisopin Kiertiburanakul, Yupin Suputtamongkol

**Affiliations:** 1 Department of Medicine, Faculty of Medicine, Siriraj Hospital, Mahidol University, Bangkok, Thailand; 2 Department of Microbiology, Faculty of Medicine, Siriraj Hospital, Mahidol University, Bangkok, Thailand; 3 Department of Immunology, Faculty of Medicine, Siriraj Hospital, Mahidol University, Bangkok, Thailand; 4 Department of Biochemistry, Faculty of Medicine, Siriraj Hospital, Mahidol University, Bangkok, Thailand; 5 Department of Medical Science, Ministry of Public Health, Nonthaburi, Thailand; 6 Department of Medicine, Faculty of Medicine, Ramathibodi Hospital, Mahidol University, Bangkok, Thailand; Cambridge University, UNITED KINGDOM

## Abstract

**Background:**

The clinical syndrome of disseminated nontuberculous mycobacterial (NTM) infection in patients who were previously healthy is now well recognized to be associated with an acquired autoantibody to Interferon gamma (Anti IFN- γ autoantibody). However, the risk factors of this syndrome remain unknown.

**Method:**

We performed an unmatched case control study among patients with NTM diseases who were diagnosed and treated at Siriraj Hospital, Bangkok, Thailand. Anti-IFN autoantibody was detected by enzyme-linked immunosorbent assay (ELISA) method. Cases were patients with NTM diseases and detectable anti IFN- γ autoantibody. Controls were randomly selected from those with undetectable anti IFN- γ autoantibody. Data from both groups including demographic data, clinical presentation, laboratory results, other risk factors and HLA genotypes were collected. Univariate and multivariate analyses were performed to identify independent risk factors for this syndrome.

**Results:**

70 cases (mean age 50 ± 11 years) and 70 controls (mean age 58 ± 18 years) were enrolled into the study. *Mycobacterial abscessus* was the most common NTM pathogen found in both groups (72.9% in cases and 41.4% in controls respectively). However, disseminated NTM disease was significantly more common in cases (92.9%) than in the controls (14.3%, p<0.001). Binary logistic regression analysis showed that previous OIs (adjusted OR14.87, 95% CI 2.36–93.86), birthplace outside Central region (adjusted OR 19.19, 95% CI 3.86–95.35), lack of comorbidities lead to immunosuppression, such as HIV infection or diabetes mellitus (adjusted OR 23.68, 95% CI 4.01–139.94), and presence of HLA DRB1*15/16 (adjusted OR 153.28, 95% CI 16.87–139.88) were independent factors associated with this syndrome.

**Conclusion:**

Patients with NTM disease associated with anti IFN- γ autoantibody are almost always previously healthy and HIV negative. Most of these patients presented with disseminated NTM disease with generalized lymphadenitis and often with reactive skin lesions. Factors associated with detectable anti IFN- γ autoantibody are HLA-DRB1 and DQB1 alleles, and history of previous OIs in patients without comorbidity that leads to immunosuppression. Further studies are needed to better understand these associations and to improve the treatment outcome.

## Background

The nontuberculous mycobacteria (NTM) are opportunistic human pathogens. Pathogenic NTM comprise of many species and strains found in the environment such as in the water and the soil. NTM disease have been increasingly diagnosed worldwide [1]. NTM diseases manifest as pneumonia, lymphadenitis, skin, soft tissue, bone and disseminated infection. Skin and soft tissue infections are almost always the result of iatrogenic or accidental inoculation of NTM caused by surgical contamination, or trauma in otherwise normal hosts. Patients with pulmonary NTM disease commonly have primary lung disorders or systemic condition that predisposed them to these infections. At the other end of the NTM spectrum, lymphadenitis and disseminated NTM disease invariably occurs in individuals with severe immunosuppression such as in advanced HIV diseases or patients with genetic defects of the Th1 pathway.

Recently, a clinical syndrome of disseminated NTM disease (with or without other opportunistic infections) in adult patients who were previously healthy is found to have acquired anti IFN- γ autoantibody [2]. Other opportunistic infections (OI) reported in this condition include nontyphoidal salmonellosis, varicella-zoster virus (VZV) infection, melioidosis, cryptococcosis and penicillosis. The clinical features of these patients are similar to those with genetic defects of the Th1 pathway in which they present with progressive or disseminated infection with mycobacteria of low virulence. This antibody recognizes functional epitopes of the cytokine IFN- γ, leading to inhibition of IFN- γ activity and exacerbation of mycobacterial disease. Apparently, the antibody presented in these patients demonstrates potent neutralizing effect. The clinical responses to antimicrobial therapy are unsatisfactory with either slow remission or frequent relapses, despite the administration of appropriate drugs and good patient compliance [3].

This syndrome is emerging as an important cause of morbidity and mortality, especially among those of Asian descent [2, 4, 5]. Although the trigger for the production of this autoantibody has not been elucidated, genetic factors are clearly involved. According to previous studies conducted in Taiwanese and Thai cohorts, this syndrome is strongly associated with HLA-DRB1 and DQB1 alleles, especially HLA-DRB1*15:01, DRB1*15:02, DRB1*16:02, DQB1*05:01 and DQB1*05:02 [6–8]. Other factors associated with this syndrome remain unknown. NTM diseases, especially disseminated NTM infection, are the most common opportunistic infection found in patients with anti IFN- γ autoantibody. This case control study aims to identify risk factors associated with this syndrome among patients with NTM diseases.

## Method

### Study design and population

We conducted an unmatched case-control study at Siriraj Hospital, Bangkok, Thailand. Study population were adult patients (more than 18 years old) with NTM diseases. All patients whom NTM was found from clinical specimens from October 2007 and April 2016 were screened. Cases were defined as patients with NTM diseases with or without other opportunistic infections, in whom autoantibody to IFN- γ were detected. All cases were followed for their infections at the Faculty of Medicine, Siriraj Hospital. Controls were randomly selected from patients with NTM diseases who had undetectable anti IFN- γ autoantibody. The study protocol was approved by the Siriraj Institutional Review Board (IRB No. Si 760/2014), and all participants provided informed written consent to participate in the study.

NTM diseases was classified as pulmonary NTM, disseminated NTM disease or localized NTM disease. Pulmonary NTM disease was defined as patients with respiratory symptoms, abnormal chest radiographic findings, and positive NTM isolation from two consecutive sputums, or one bronchial wash or bronchoalveolar lavage sample as per the ATS/IDSA 2007 recommendations [9]. Disseminated NTM disease was defined as patients who had NTM isolated from blood/bone marrow culture or patient with NTM isolated from sterile site and at least two non-contiguous organ involvement. Localized NTM disease was defined as patients with NTM isolated from one specific site or organ such as lymph node, skin and soft tissue, bone and joint.

### Procedures

At enrollment, complete histories were obtained and physical examinations with routine clinical laboratory tests were performed for both cases and controls. Demographic data, place of birth, detailed characteristics of their infections such as site of infection, causative organism, clinical courses and outcome of treatment were also recorded. Clinical presentations prior to the diagnosis of NTM diseases and comorbidities that could lead to immunosuppression, including diabetes mellitus (DM), Human Immunodeficiency Virus (HIV) infection, autoimmune or neoplastic disease, stem cell or solid organ transplantation or use of immunosuppressive agents within 3 months, in both cases and controls were reviewed from the medical records. Previous diagnosis of OI included nontyphoidal salmonellosis, VZV infection, tuberculosis, NTM diseases, cryptococcosis, endemic mycoses and melioidosis. Reactive skin diseases associated with this syndrome were diagnosed as Sweet syndrome, acute generalized exanthematous pustulosis (AGEP), pustular psoriasis or erythema nodosum [10]. Anti IFN- γ autoantibody in the serum was measured by an enzyme-linked immunosorbent assay (ELISA) as previously described[8]. The result is considered positive if the optical density (OD) is at least 1. This cut-off was selected according to the in-house data comparing between a group of patients and more than 100 normal controls.

Baseline laboratories included complete blood count, plasma urea, creatinine, liver function test, antinuclear antibody (ANA), erythrocyte sedimentation rates (ESR), C-reactive protein (CRP), anti-HIV antibody, Immunoglobulin G (IgG), CD4, CD8 and Human leukocyte antigens (HLA). HLA genotyping was done with the next generation sequencing platform using GS Junior System (Roche/454 Life science, Branford, USA) as previously described [8]. Data were recorded on standardized case record forms.

### Sample size calculation

Based on previous studies from Taiwan and Thailand [6, 8], only specific HLA class II genotypes were associated with this clinical syndrome. There was no data about other clinical risk factors of this syndrome. In this study we assumed that the prevalence of other risk factors among cases is at least 25%, with OR of at least 4, a two-sided alpha error of 0.05, and 80% power, the sample size was calculated to be 60 patients per group. Thus we intended to enroll at least 60 cases and 60 controls.

### Statistical analysis

Descriptive statistics were used to demonstrate patient baseline characteristics. Categorical data was presented as frequency and continuous data was presented as the mean with standard deviation or median with range, depending on the distribution of the data. Chi-square or Fishers exact test and independent t-test or Mann-Whitney U test were used for the univariate analysis compared between case and control as appropriate.

The association between various possible risk factors and this clinical syndrome was calculated as an odds ratio (OR) and 95% confidence interval (CI). Independent risk factors were identified by binary logistic regression model to adjust for possible risk factors, defined as variables that revealed P<0.05 in univariate analysis.

## Results

There were 87 patients with detectable anti IFN- γ autoantibody associated with OIs during the study period, and 70 of them had NTM diseases. Median of Anti-IFN gamma autoantibody among case group was 3.64 (range 1.32–4.61). We randomly selected 70 patients with NTM disease and negative anti IFN- γ autoantibody. Mean age of cases was 50 ± 11 years while mean age of controls was 58 ± 18 years. The sex distribution among cases and controls was similar. Birthplace of the cases were equally distributed from the central, north and northeast regions of Thailand, but for the controls, most of them were from the central region. Comorbidities among cases were significantly lower than among controls (4 patients, 5.7% VS 24 patients, 34.3% respectively, p<0.001). Comorbidities among cases included diabetes mellitus (DM) in 2 patients and systemic lupus erythematosus (SLE) in 2 patients. HIV infection was found in 11 patients from the control group but none from the case group. Other comorbidities among the controls included DM in 7 patients, SLE in 4 patients, solid malignancy in 2 patients, and solid organ transplantation in 2 patients.

Approximately half of the cases (33, 47.1%) had history of OIs prior to the detection of anti IFN- γ autoantibody. Common OIs among cases included nontyphoidal salmonellosis (17 patients, 24.3%) and NTM disease (14 patients, 20%). Other OIs included tuberculosis (5 patients, 7.1%), VZV infection (4 patients, 5.7%), fungal infection (4 patients, 5.7%) and melioidosis (2 patients, 2.9%). HLA analysis was performed in 63 cases and 57 controls. Results showed that HLA-DRB1*15:02, DRB1*16:02, DQB1*05:01 and DQB1*05:02 were significantly more common in the cases compared to in the controls. HLA-DRB*16:01 and DRB*16:09 were found only in the case group. Details of demographic data, distribution of birthplace and HLA genotype distribution between cases and controls are shown in Table 1.

**Table 1:** Baseline characteristic and genetic studies.

Variable	Case (n = 70)	Control (n = 70)	p-value
**Female, n (%)**	40 (57.1)	37 (52.9)	0.734
**Age, yrs (Mean±SD)**	50 ± 11	58 ± 18	0.003
**Birthplace, n (%)**			< 0.001
Central	21 (30)	51 (72.9)	
North	16 (22.9)	1 (1.4)	
Northeast	18 (25.7)	3 (4.3)	
West	10 (14.3)	6 (8.6)	
South	5 (7.1)	9 (12.9)	
**Comorbidities**			
Yes**, n (%)**	4 (5.7)	24 (34.3)	< 0.001
**Previous OIs**			
Yes**, n (%)**	33 (47.1)	11 (15.7)	< 0.001
**HLA studies** **HLA-DRB1, n (%)**			
DRB1*15	42 (66.7)	21 (36.8)	0.001
DRB1*15:01	18 (28.6)	14 (24.6)	0.620
DRB1*15:02	24 (38.1)	8 (14.0)	0.003
DRB1*16	43 (68.3)	3 (5.3)	<0.001
DRB1*16:01	4 (6.3)	0 (0)	0.121
DRB1*16:02	36 (57.1)	3 (5.3)	<0.001
DRB1*16:09	4 (6.3)	0 (0)	0.121
DRB1*15 or 16	61 (96.8)	24 (42.1)	<0.001
**HLA-DQB1, n (%)**			
DQB1*05	59 (93.7)	22 (38.6)	< 0.001
DQB1*05:01	25 (39.7)	9 (15.8)	<0.001
DQB1*05:02	42 (66.7)	16 (28.1)	<0.001
DQB1*05:03	1 (1.6)	2 (3.5)	0.607

The most common clinical presentation in the cases (92.9%) was disseminated NTM disease. All of them had bilateral cervical or generalized lymphadenopathies. Hepatomegaly and splenomegaly were noticed in 20% and 11.4% respectively. Most causative NTM pathogens were identified or isolated from lymph node, blood or bone marrow. Approximately two-thirds of the cases (44 patients) also developed reactive skin lesions associated with active NTM diseases. Common reactive skin lesions included Sweet syndrome (26 patients, 37.1%), AGEP (14 patients, 20%), erythema nodosum (10 patients, 14.3%), urticarial-like, exfoliative dermatitis and pustular psoriasis-like lesion (1 patient each). Reactive arthritis was also detected in 6 patients (8.6%)

Localized NTM diseases among the controls included pulmonary NTM disease (25 patients, 35.7%), cutaneous NTM disease (13 patients, 18.6%), nosocomial NTM disease (10 patients, 14.3%), bone and joint involvement (8 patients, 11.4%) and other NTM diseases (4 patients, 5.7%). Lymph node involvement without disseminated disease was found in only one patient. Among control group, disseminated NTM diseases were diagnosed in 10 patients (14.3%). Seven of them were HIV infected, 1 patient had SLE and 2 patients had no comorbidity. Only one patient from the controls developed reactive skin reaction (erythema nodosum).

Rapidly growing mycobacteria were the most common causative NTM pathogen in both groups, but the proportion of rapid grower was significantly higher among the cases (81.4%) than among the controls (61.4%, p 0.009). *Mycobacterium abscessus* was the most commonly found NTM pathogen in both groups. Other common causative NTM pathogens identified in both cases and controls included *M*. *avium intracellulare* complex (MAC) and *M*. *fortuitum*. Three patients in case group had mixed NTM infections.

Anemia, leukocytosis, eosinophilia and thrombocytosis were more common among the cases than among the controls. Acute phase reactant markers (ESR and CRP) were also significantly higher in the cases than in the controls. The case patients generally had higher globulins and lower albumin levels compared to the controls. ANA was frequently detected in both groups, of which fine speckle pattern was the most common type. CD4 count was lower in the controls than in the cases. Baseline renal function test results were comparable between both groups. Detail of pathogens, site of isolation, and results of other laboratory investigations are shown in Table 2.

**Table 2:** Laboratory investigations.

Characteristic, n (%)	Case (n = 70)	Control (n = 70)	p-value
**Species of NTM**			
*M*. *abscessus*	51 (72.9)	29 (41.4)	< 0.001
*MAC*	*8 (11*.*4)*	*14 (20)*	0.164
*M*. *fortuitum*	7 (10)	12 (17)	0.217
*M*. *kansasii*	1 (1.4)	6 (8.6)	0.116
*M*. *simiae*	1 (1.4)	2 (2.9)	1.000
*M*. *haemophilum*	1 (1.4)	1 (1.4)	1.000
*M*. *scrofulaceum*	1 (1.4)	0	0.493
Others*	0	3 (4.3)	
Unidentified	3 (4.3)	2 (2.9)	
**Sites of positive C/S**			
Lymph node	51 (72.9)	3 (4.3)	< 0.001
Blood or bone marrow	23 (32.9)	10 (14.3)	0.010
Sputum	9 (12.9)	26 (37.1)	0.001
Skin or pus	6 (8.6)	13 (18.6)	0.084
Pleural fluid	1 (1.4)	2 (2.1)	1.000
Synovial fluid	1 (1.4)	9 (12.9)	0.009
Lung tissue	1 (1.4)	2 (2.1)	1.000
Bowel	1 (1.4)	1 (1.4)	1.000
Cardiac valve	1 (1.4)	0	0.493
Others**	0	9 (12.9)	
**Blood tests, median (min-max)**			
Hematocrit, %	30.7 (13.5–44.6)	36.2 (18.7–53.1)	0.011
White blood cell, /mm^3^	20,020 (721–56,680)	7,365 (2,150–25,350)	< 0.001
Total eosinophil, /mm^3^	802 (23–6,192)	120 (0–1,039)	< 0.001
Platelet, x100/mm^3^	377.5 (43–953)	287.5 (50–543)	< 0.001
ESR, mm/hr	81 (5–123)	43 (2.7–119)	0.009

* *M*. *marinum* (2 patients), *M*. *chelonae* (1 patient)

** Other sites with positive culture included bone (3 patients), Stool (2 patients), vascular graft (1 patient), liver (1 patient) and eye (2 patient)

Results of binary logistic regression analyses are shown in Table 3. Lack of comorbidities, birthplace, previous OIs, and presence of HLA-DRB1*15 or 16 and HLA-DQB1*05:01 or 05:02 were independent factors for the development of anti IFN- γ autoantibody.

**Table 3:** Results of multivariate analyses.

Factors	Crude OR	95%CI	Adjusted OR	95% CI	p-value
Age	0.97	2.69–12.97	0.96	0.92–1.01	0.088
Previous OIs	4.78	2.16–10.61	14.87	2.36–93.86	0.004
Birthplace outside central region	8.61	2.80–26.48	19.19	3.86–95.35	<0.001
Lack of comorbidities	6.26	3.01–13.05	23.68	4.01–139.94	<0.001
HLA alleles*	41.94	9.33–188.59	153.28	16.87–139.88	<0.001

* HLA alleles: Presence of HLA-DRB1*15 or 16

## Discussion

It has been well recognized that NTM diseases were the most common OIs associated with an acquired anti IFN- γ autoantibody. Finding among the cases showed that this syndrome was equally diagnosed in men and women. The controls or NTM patients without detectable anti IFN- γ autoantibody also revealed similar gender distribution. In this study, we demonstrated that among patients with NTM diseases, specific HLA class II alleles (DRB*15, DRB1*16, DQB*05:01 and DQB1*05:02), comorbidities, history of previous OI and birthplace were significantly associated with an acquired anti IFN- γ autoantibody. However, geographical distribution bias cannot be excluded because the study hospital is located in the central region, and laboratories to confirm the diagnosis of both NTM and anti IFN- γ autoantibody in Thailand are not generally available elsewhere. Patients with disseminated NTM diseases, which represented the majority of the case group, were likely to be referred or to seek further medical services at large university hospital. Therefore, suspected patients with this clinical syndrome throughout the country were referred to our hospital for the diagnosis and treatment. By contrast, the patients with localized NTM infection in the control group were mainly from the region of the hospital service area.

Results of this study also confirmed previous clinical observation that the patients with anti IFN- γ autoantibody were almost always previously healthy and HIV negative. They frequently presented with disseminated NTM disease, with generalized lymphadenitis and associated reactive skin lesions, especially Sweet syndrome and AGEP. In contrast to the case group, disseminated NTM diseases in control group were commonly found among HIV infected patients, and only one patient developed reactive skin lesion.

History of nontyphoidal salmonellosis was also significantly associated with NTM diseases among the patients with detectable anti IFN- γ autoantibody. Although a patient with this clinical syndrome was first reported in 2006 [4, 11], it was not recognized until recently that acquired anti IFN- γ autoantibody is associated with *Salmonella* infection [12]. History of recurrent NTM diseases was prevalent among the cases, partly due to a lack of awareness for this clinical syndrome prior to the year 2012 [2]. Since then, anti IFN- γ autoantibody have been routinely tested in non HIV-infected patients with the first episode of NTM disease in Thailand. As a result, history of previous NTM diseases could become less common in the future when this condition is widely recognized and quickly detected.

The prevalence of NTM diseases has increased worldwide, but the epidemiology of NTM disease in Thailand is different from the previous report [13]. Rapidly growing mycobacteria were the most common cause of NTM diseases found in this study. Although *M*. *abscessus* was the most common pathogen found in both groups, it was significantly more common among cases compared to the controls. There is no clear explanation for this finding. MAC was reported to be the most common causative NTM pathogen among HIV-infected patients [14]. We found MAC infection in 6 out of 7 HIV-infected patients who had disseminated NTM diseases.

Several laboratory findings among the case were markedly different from the control group. While some parameters, such as anemia and low serum albumin would reflect chronic systemic infection rather than unique characteristics of the patients with anti IFN- γ autoantibody, it is unclear whether the high serum globulin were caused by persistent infection or by increased production of autoantibody. Long-term follow up of these patients are required to better understand those laboratory changes. Similar to previous reports, our study confirmed that specific HLA class II alleles were strongly associated with this clinical syndrome [6–8]. In contrast to our previous report, we did not see the significant association between HLA-DRB1*15:01 and anti IFN- γ autoantibody when compared to NTM infected patients without autoantibody. This was likely explained by the high allele frequency of HLA-DRB1*15:01 in the control group. In fact, NTM patients without anti IFN- γ autoantibody represented much higher HLA-DRB1*15:01 allele frequency than Thai general population [15]. This frequency difference was not observed in HLA-DRB1*15:02, DRB1*16:02, DQB1*05:01 and DQB1*05:02. There was no previous report on HLA-DRB1 and rapidly growing mycobacterial infection. However, HLA-DRB1*15:01 was found to be significantly associated with MAC infection and leprosy [16, 17]. So It is possible that HLA-DRB1*15:01 is associated with NTM diseases regardless of autoantibody status. This finding could elaborate the clinical observation that NTM diseases were the most common infection in this syndrome.

Because NTM disease was used as the major determinant for this case-control study, the clinical and laboratory parameters significantly associated with detectable anti IFN- γ autoantibody in this study may not be applicable to patients presented with other OIs. The geographical distribution bias in the control group may overestimate the influence of this factor. We also used only clinical data obtained at the time of diagnosis for analysis. Therefore, the result may not entirely reflect the clinical picture of this syndrome which is often fluctuating. Nevertheless, our study is the largest cohort on clinical picture of patients with anti IFN- γ autoantibody reported to date [2, 5, 12]. The result of this study affirms the clinical observation that this immunodeficiency syndrome should be considered in previously healthy patients with NTM diseases who has history of previous OI. Anti IFN- γ autoantibody should be tested for definite diagnosis so appropriate management could be given. Additional studies are warranted to provide a better understanding on the clinical courses of this syndrome.
